# Combination therapy with nutritional vitamin D and calcimimetics in secondary hyperparathyroidism: a mechanism-based approach to restoring PTH regulation

**DOI:** 10.3389/fnut.2026.1871009

**Published:** 2026-07-07

**Authors:** Chien-Lin Lu, Yi-Chou Hou, Chia-Chao Wu, Te-Chao Fang, Cai-Mei Zheng, Kuo-Cheng Lu

**Affiliations:** 1 Division of Nephrology, Department of Internal Medicine, Fu Jen Catholic University Hospital, Fu Jen Catholic University, New Taipei City, Taiwan; 2 School of Medicine, College of Medicine, Fu Jen Catholic University, New Taipei City, Taiwan; 3 Division of Nephrology, Department of Internal Medicine, Cardinal-Tien Hospital, New Taipei City, Taiwan; 4 Division of Nephrology, Department of Internal Medicine, Tri-Service General Hospital, National Defense Medical Center, Taipei, Taiwan; 5 Division of Nephrology, Department of Internal Medicine, School of Medicine, College of Medicine, Taipei Medical University, Taipei, Taiwan; 6 Division of Nephrology, Department of Internal Medicine, Taipei Medical University Hospital, Taipei Medical University, Taipei, Taiwan; 7 Taipei Medical University-Research Center of Urology and Kidney, Taipei Medical University, Taipei, Taiwan; 8 Division of Nephrology, Department of Internal Medicine, Shuang Ho Hospital, Taipei Medical University, New Taipei City, Taiwan; 9 TMU Research Center of Urology and Kidney, Taipei Medical University, Taipei, Taiwan; 10 Division of Nephrology, Department of Medicine, Taipei Tzu Chi Hospital, Buddhist Tzu Chi Medical Foundation, New Taipei City, Taiwan

**Keywords:** calcimimetics, fibroblast growth factor 23, nutritional vitamin D, parathyroid hormone, secondary hyperparathyroidism

## Abstract

Secondary hyperparathyroidism (SHPT) in chronic kidney disease (CKD) is commonly interpreted as a compensatory response to disturbances in mineral metabolism, yet this view does not fully account for the biochemical instability and treatment resistance observed in advanced disease. Current therapeutic strategies primarily target individual regulatory pathways. Calcimimetics suppress parathyroid hormone (PTH) secretion through calcium-sensing receptor (CaSR) activation, whereas active vitamin D analogs reduce PTH synthesis via vitamin D receptor (VDR) signaling but are frequently associated with increased mineral load and elevated fibroblast growth factor 23 (FGF-23). Nutritional vitamin D (NVD) restores circulating 25-hydroxyvitamin D [25(OH)D] and supports tissue-level activation of vitamin D pathways without substantial increases in calcium or phosphate. When combined with calcimimetics, NVD provides complementary modulation of PTH regulation through distinct but interacting mechanisms. Experimental and clinical evidence suggests that this combination may enhance responsiveness of parathyroid tissue and contribute to improved biochemical control. At the system level, this approach has been associated with reduced PTH variability, relatively stable calcium balance, stabilization or modest reduction in phosphate, and reduction in FGF-23 compared with active vitamin D–based strategies. However, current evidence remains largely based on surrogate biochemical endpoints. The Evaluation Of Cinacalcet HCl Therapy to Lower Cardiovascular Events (EVOLVE) trial, the largest Randomized Controlled Trial (RCT) of cinacalcet in dialysis patients, demonstrated no significant reduction in the primary composite endpoint of all-cause mortality or major nonfatal cardiovascular events in the intention-to-treat analysis despite consistent biochemical improvements, and the extent to which combination calcimimetic-based therapy translates into improved long-term clinical outcomes therefore remains uncertain.

## 1 SHPT as a disorder of integrated PTH regulation

Secondary hyperparathyroidism (SHPT) in chronic kidney disease (CKD) is traditionally viewed as a compensatory endocrine response to disturbances in calcium, phosphate, and vitamin D metabolism. While this framework explains the initiation of parathyroid hormone (PTH) elevation, it does not fully account for the persistent dysregulation, treatment resistance, and biochemical instability observed in advanced disease. A more physiologically coherent interpretation is to consider SHPT as a disorder of dysregulated dual-axis control of PTH, involving both rapid hormone secretion and longer-term transcriptional regulation. Within this model, PTH excess reflects not only sustained external stimuli—such as phosphate retention, hypocalcemia, and reduced calcitriol—but also intrinsic alterations in parathyroid signaling, receptor expression, and glandular architecture. This conceptual shift has therapeutic implications, suggesting that effective treatment requires restoration of coordinated regulation across both axes rather than isolated suppression of PTH through a single pathway.

### 1.1 Secretory and transcriptional regulation of PTH

Physiological regulation of PTH depends on two complementary but mechanistically distinct control systems. The first is a rapid secretory axis mediated by the calcium-sensing receptor (CaSR), through which extracellular calcium directly regulates PTH release from parathyroid cells. When extracellular calcium rises, CaSR activation suppresses PTH secretion; when calcium falls, this inhibitory signal is reduced, permitting increased PTH release (1, 2). The second is a slower transcriptional axis mediated by calcitriol–vitamin D receptor (VDR) signaling. Calcitriol binds to VDR and represses PTH gene transcription through vitamin D response elements in the PTH promoter, thereby limiting PTH synthesis rather than immediate secretion (2, 3).

These two regulatory axes are functionally interconnected. VDR activation can increase CaSR expression through vitamin D response elements within the CaSR gene, thereby enhancing parathyroid sensitivity to extracellular calcium (4). This interaction supports coordinated regulation of both secretion and transcription.

In CKD, both axes are progressively impaired. Reduced calcitriol availability, driven by impaired renal 1α-hydroxylase (CYP27B1) activity and fibroblast growth factor 23 (FGF-23)-mediated suppression, weakens VDR-dependent repression of PTH transcription (5, 6). At the same time, CaSR expression is reduced in hyperplastic parathyroid tissue, and hyperphosphatemia may directly antagonize CaSR activity, shifting the calcium–PTH relationship toward persistent secretion (7, 8). Together, these abnormalities support dual-axis dysregulation of PTH in CKD.

### 1.2 Parathyroid remodeling and loss of regulatory responsiveness

Sustained dysregulation of PTH in CKD is associated with progressive structural remodeling of the parathyroid glands. Early stages of SHPT are characterized by diffuse, polyclonal hyperplasia, which may evolve into nodular hyperplasia with increased proliferative activity and more autonomous behavior under persistent uremic stimulation (9). Nodular glands exhibit higher proliferative indices, including increased Ki-67 expression and activation of cell-cycle pathways such as cyclin D1, supporting their growth advantage and reduced dependence on external regulatory signals (10, 11). Functionally, these glands display a rightward shift in the calcium–PTH set point, indicating reduced sensitivity to calcium-mediated suppression (12).

A central feature of this remodeling process is the downregulation of both CaSR and VDR. Multiple studies have demonstrated reduced expression of these receptors in hyperplastic parathyroid tissue, particularly in nodular lesions (7, 13, 14). Loss of CaSR reduces responsiveness to extracellular calcium, while reduced VDR weakens transcriptional repression of PTH. In addition, decreased VDR expression has been associated with reduced expression of cell-cycle inhibitors such as p21 and p27, linking receptor loss to increased cellular proliferation (15). Experimental data further suggest that high phosphate exposure and epigenetic modifications, including promoter hypermethylation, may contribute to suppression of CaSR and VDR expression (16, 17).

These structural and molecular changes are accompanied by progressive loss of regulatory responsiveness. As SHPT advances, additional pathways—including FGF-23 signaling through FGFR1–Klotho—become impaired, further reducing feedback control of PTH (13). Clinically, nodular hyperplasia is associated with reduced responsiveness to vitamin D–based therapies and may necessitate parathyroidectomy in advanced cases (12, 15).

FGF23 signaling in the parathyroid gland follows a stage-dependent pattern that is relevant to the therapeutic positioning of NVD. In normal and early-stage parathyroid tissue, FGF23 acts through the parathyroid FGFR1–Klotho complex to suppress PTH secretion and stimulate parathyroid CYP27B1 expression, effects opposite to its suppressive actions on renal CYP27B1 (18, 19). This parathyroid CYP27B1 stimulation is substrate-dependent, generating local calcitriol only when circulating 25(OH)D is adequate. As SHPT advances, however, FGFR1 and Klotho expression in hyperplastic parathyroid glands declines progressively, and FGF23 signaling shifts from a PTH-suppressive to a proliferative stimulus (20, 21). The PTH-suppressive and CYP27B1-stimulatory effects of FGF23 in the parathyroid are therefore operative in early disease but lost, and reversed, as hyperplasia progresses.

The temporal sequence of these regulatory disturbances reflects a pattern more consistent with hysteresis than with linear endocrine feedback. In early CKD, phosphate retention is initially buffered by FGF-23 elevation without PTH involvement; concurrent calcium retention may further delay the parathyroid response by maintaining extracellular calcium within the normal range. PTH elevation therefore emerges as a late event reflecting a fundamentally altered regulatory state rather than a proportionate acute compensatory response (22). Once established, SHPT represents a dysregulated steady state in which structural and molecular remodeling, including progressive CaSR and VDR downregulation, nodular hyperplasia, and altered set-point calcium sensitivity, cannot be fully reversed by biochemical suppression alone.

### 1.3 System-level consequences within the CKD-MBD network

Parathyroid dysfunction in CKD occurs within the broader framework of CKD–mineral–bone disorder (CKD-MBD), an integrated endocrine network involving phosphate, FGF-23, Klotho, vitamin D metabolism, and PTH rather than an isolated parathyroid disorder (23). Declining kidney function leads to phosphate retention, which stimulates FGF-23 production and suppresses calcitriol synthesis, contributing to increased PTH levels (24). Although these compensatory mechanisms may initially maintain near-normal mineral balance, progressive Klotho deficiency impairs FGF-23 signaling, resulting in simultaneous elevation of FGF-23 and PTH and disruption of feedback regulation (25, 26).

Within this dysregulated network, abnormalities in PTH extend beyond bone metabolism. Sustained PTH elevation is associated with renal osteodystrophy, while skeletal resistance to PTH further complicates the relationship between circulating PTH and bone turnover (27). In parallel, phosphate retention, PTH, and FGF-23 contribute to vascular calcification and cardiovascular remodeling and are associated with increased cardiovascular risk and mortality in CKD populations (28–30). Importantly, both markedly elevated and excessively suppressed PTH levels are associated with adverse outcomes, suggesting that regulatory instability, rather than absolute PTH levels alone, is clinically relevant (31).

## 2 Limitations of current therapies in restoring PTH regulation

Current therapies for SHPT remain limited by their inability to restore coordinated regulation of PTH. Most interventions act on a single regulatory pathway, resulting in partial biochemical control that is often accompanied by new disturbances in mineral metabolism.

### 2.1 Transcription-focused therapy: efficacy and mineral trade-offs

Active vitamin D analogs suppress PTH primarily through VDR-mediated transcriptional repression. Binding of calcitriol to VDR reduces PTH gene expression and inhibits parathyroid cell proliferation, making this approach effective in lowering PTH levels and controlling glandular hyperplasia (32).

However, VDR activation is not confined to the parathyroid gland. Active vitamin D increases intestinal absorption of calcium and phosphate. In SHPT, sustained PTH excess already drives bone mineral mobilization through osteoclastic activation; active vitamin D, by simultaneously increasing intestinal calcium and phosphate absorption, further compounds this mineral load rather than independently initiating it (33, 34). These effects are associated with hypercalcemia, hyperphosphatemia, and increased calcium–phosphate product, particularly at higher doses (32, 34).

These metabolic changes reflect a key limitation of transcription-focused therapy: systemic activation of the VDR axis without restoration of upstream regulatory balance. As SHPT progresses, dose escalation is often required, further increasing mineral load and potentially contributing to vascular calcification and long-term safety concerns (35).

A further mechanistic limitation of transcription-focused therapy relates to progressive loss of VDR expression in hyperplastic parathyroid tissue. In CKD, the three principal drivers of parathyroid cell growth—low calcium, high phosphate, and vitamin D deficiency—each induce parathyroid transforming growth factor-α (TGF-α), which activates the epidermal growth factor receptor (EGFR) and promotes synthesis of liver-enriched inhibitory protein (LIP), the dominant-negative isoform of CCAAT/enhancer-binding protein-β (C/EBPβ). LIP antagonizes C/EBPβ transactivation of the VDR gene promoter, resulting in progressive VDR downregulation that reaches up to 80% reduction in VDR mRNA in nodular hyperplasia—the most severe and calcitriol-resistant form of SHPT (36, 37). This LIP-driven VDR silencing renders parathyroid tissue progressively less responsive to calcitriol regardless of the dose administered, and represents a key reason why active vitamin D therapy alone becomes insufficient as SHPT advances. Importantly, experimental evidence suggests that correction of vitamin D deficiency through 25-hydroxyvitamin D supplementation synergizes with calcitriol—whether through intracrine CYP27B1-mediated conversion of 25(OH)D or through direct occupancy of the alternative VDR pocket (VDR-AP) by 25(OH)D itself, or both—to induce parathyroid C/EBPβ expression, thereby counteracting LIP-mediated VDR suppression and partially restoring parathyroid responsiveness to active vitamin D (38). This observation provides a mechanistic basis for combining nutritional vitamin D (NVD) with other therapeutic modalities rather than relying on active vitamin D dose escalation alone.

### 2.2 Secretion-focused therapy: effective suppression with incomplete control

Calcimimetics lower PTH by activating CaSR on parathyroid cells, enhancing sensitivity to extracellular calcium and suppressing hormone secretion. This produces a rapid reduction in circulating PTH levels and avoids the hypercalcemic effects associated with active vitamin D analogs (39). In dialysis populations, meta-analyses demonstrate consistent reductions in PTH and serum calcium, supporting their role as secretion-focused therapy in SHPT management (40, 41).

However, calcimimetic therapy alone does not fully restore PTH regulation. By reducing bone resorption and calcium release, and through CaSR activation in the intestine and kidney, calcimimetics frequently lower serum calcium and may induce hypocalcemia (42). Treatment has also been associated with reductions in circulating 1,25(OH)_2_D and increases in serum phosphate, indicating that VDR-mediated transcriptional control is not restored and may be further compromised (42, 43). These changes may promote counter-regulatory PTH stimulation.

As a result, PTH secretion may be acutely suppressed while long-term regulation remains unstable. In non-dialysis CKD, this is reflected in relatively modest and less durable PTH reductions with monotherapy (44). Calcimimetics also do not correct the high prevalence of 25(OH)D deficiency in CKD, leaving a key component of vitamin D–dependent signaling unaddressed (45, 46).

Critically, the biochemical improvements achieved with calcimimetic therapy have not translated into demonstrated benefits on patient-level outcomes. The EVOLVE trial—the largest RCT of cinacalcet in dialysis patients—demonstrated no statistically significant reduction in the primary composite endpoint of all-cause mortality or major nonfatal cardiovascular events in the intention-to-treat analysis (HR: 0.93, 95% CI: 0.85–1.02), despite consistent improvements in PTH, serum calcium, and serum phosphorus (47). Although adjusted analyses suggested a modest 12%−13% relative risk reduction and sensitivity analyses accounting for non-adherence indicated stronger on-treatment effects, the pre-specified primary analysis was negative. A Cochrane systematic review encompassing 18 RCTs and 7,446 participants similarly found little or no effect of cinacalcet on all-cause mortality (RR: 0.97, 95% CI: 0.89–1.05), with uncertain effects on cardiovascular mortality despite consistent biochemical improvements (48), indicating that serum PTH and calcium are not sufficiently validated surrogates for hard patient outcomes in calcimimetic trials.

The hypocalcemia risk associated with calcimimetic therapy is not fully corrected by concurrent active vitamin D analog use. Whether NVD mitigates or compounds calcimimetic-induced hypocalcemia remains to be directly quantified. A network meta-analysis of 21 RCTs including 4,653 hemodialysis patients demonstrated that the combination of calcimimetics with active vitamin D analogs still produced a net reduction in serum calcium relative to placebo (MD: −5.83 mg/dl, 95% CI: −9.73 to −1.93) (49). These findings indicate that the available evidence pertains specifically to active vitamin D analog combinations and should not be extrapolated to NVD-based regimens.

### 2.3 Single-pathway targeting in a multi-regulatory disorder

Current therapies for SHPT predominantly target either transcriptional or secretory regulation of PTH rather than both simultaneously. Active vitamin D analogs suppress PTH synthesis through VDR-mediated mechanisms, whereas calcimimetics reduce PTH secretion via CaSR activation (32, 43). While both approaches lower PTH, neither alone restores coordinated regulation across these control systems.

This limitation is further influenced by progressive parathyroid remodeling, which reduces expression of CaSR and VDR and diminishes responsiveness to calcium and vitamin D signaling (7, 50). Clinically, this is reflected in variable biochemical responses and incomplete or unstable PTH control despite treatment (39). These observations suggest a gap between biochemical suppression of PTH and restoration of physiological regulation. This gap provides a rationale for therapeutic strategies that engage both regulatory pathways and incorporate restoration of vitamin D substrate availability.

## 3 Nutritional vitamin D as a substrate-level therapeutic strategy

NVD should not be regarded solely as replacement therapy for deficiency, but as a substrate-level intervention that restores the biological context of PTH regulation. In CKD, disruption of vitamin D metabolism reflects not only reduced calcitriol production but also depletion of circulating 25(OH)D, which limits both systemic and local vitamin D signaling. By repleting substrate availability, NVD enables intracrine and paracrine activation of vitamin D pathways, supporting VDR-dependent regulation without imposing supraphysiologic systemic exposure. Its contribution is therefore more accurately characterized as preserving endogenous control over vitamin D activation rather than substituting for PTH action or serving as a primary PTH-lowering agent. In advanced CKD, where LIP-driven VDR silencing and structural parathyroid remodeling constrain the biological impact of substrate-dependent pathways, this contribution is likely to be more limited, and this should be acknowledged in interpreting the clinical relevance of combination therapy in late-stage disease.

### 3.1 Restoration of intracrine vitamin D signaling

In CKD, both circulating calcitriol and 25(OH)D levels decline, impairing systemic endocrine signaling and local vitamin D–dependent regulation within target tissues. Parathyroid tissue expresses both 1α-hydroxylase and VDR, allowing local conversion of circulating 25(OH)D to calcitriol and subsequent suppression of PTH transcription (51). In addition, extra-renal tissues retain 1α-hydroxylase activity, supporting a model in which circulating 25(OH)D serves as substrate for tissue-specific vitamin D activation (45).

Repletion of 25(OH)D through NVD may restore this intracrine signaling capacity. Experimental and clinical data suggest that adequate 25(OH)D availability supports local VDR activation and contributes to modest suppression of PTH without substantially increasing serum calcium or phosphate at typical doses (52). However, the quantitative contribution of local 1α-hydroxylase activity within the parathyroid gland remains incompletely defined (51). These findings support a model in which NVD primarily restores substrate availability for tissue-level signaling rather than directly driving systemic VDR activation.

The inhibitory effect of FGF-23 on 1α-hydroxylation is directed primarily at renal CYP27B1 and does not apply to parathyroid intracrine vitamin D activation. In hyperplastic parathyroid tissue, CYP27B1 expression is upregulated approximately 10-fold relative to normal glands, while 24-hydroxylase (CYP24A1) is simultaneously downregulated to approximately one-tenth of normal levels, maximizing local calcitriol generation capacity and minimizing its intracellular catabolism (9). Calcimimetic therapy further increases parathyroid CYP27B1 activity by approximately 42%, amplifying the local demand for 25(OH)D substrate. This state of increased enzymatic capacity for local calcitriol synthesis alongside progressively impaired substrate delivery due to declining megalin expression and uremic barriers provides the mechanistic basis for NVD supplementation as a substrate-level intervention that supports parathyroid intracrine activation independently of FGF-23-mediated suppression of renal CYP27B1.

### 3.2 Formulation-dependent differences in substrate delivery

The therapeutic use of NVD requires pharmacological distinction between its principal forms. Cholecalciferol and ergocalciferol are prodrugs that require hepatic 25-hydroxylation via vitamin D 25-hydroxylase (CYP2R1) and sterol 27-hydroxylase (CYP27A1) before entering the active vitamin D metabolic pathway, whereas calcifediol is a pre-formed 25(OH)D compound that bypasses this conversion step entirely. This distinction has clinically significant consequences in CKD, where hepatic 25-hydroxylase activity is reduced in both CKD and obesity (53, 54), and where adipose-related dilution further limits the bioavailability of fat-soluble cholecalciferol and ergocalciferol (55, 56). Real-world data from European and US non-dialysis CKD cohorts demonstrate that more than 50% of patients treated with cholecalciferol or ergocalciferol failed to achieve serum 25(OH)D ≥30 ng/ml, and only 7.3%−7.5% achieved levels ≥50 ng/ml (57). However, these data derive from investigators affiliated with OPKO Health, Inc. (OPKO), the manufacturer of extended-release calcifediol (Rayaldee), and have not been independently replicated; they should therefore be interpreted with appropriate caution. Nonetheless, the direction of these findings is consistent with meta-analytic evidence showing only modest and highly variable increases in serum 25(OH)D with cholecalciferol or ergocalciferol supplementation in non-dialysis CKD (52, 58). In a head-to-head randomized trial in CKD stages 3–5, cholecalciferol raised serum 25(OH)D significantly more than ergocalciferol (mean +45 vs. +31 ng/ml; *p* < 0.01), though both showed declining levels after treatment cessation, underscoring the need for ongoing maintenance (59).

Two physiological barriers further limit substrate delivery in CKD. First, hepatic 25-hydroxylation of cholecalciferol and ergocalciferol is impaired in CKD and obesity, reducing the reliability of these compounds in achieving target serum 25(OH)D concentrations. Second, uptake of circulating vitamin D-binding protein (DBP)-bound 25(OH)D into parathyroid chief cells via megalin [low-density lipoprotein receptor-related protein 2 (LRP2)]-mediated endocytosis is hypothesized to be impaired in the uremic environment, by analogy with the well-documented reduction of megalin function in proximal tubular cells; direct evidence in parathyroid chief cells is currently lacking, although, if present, such a barrier could limit cellular substrate availability even when circulating 25(OH)D levels are adequate. Calcifediol bypasses the hepatic conversion step but remains dependent on megalin-mediated cellular uptake and is therefore subject to the second barrier. Cholecalciferol, by contrast, may enter parathyroid chief cells directly through passive membrane diffusion by virtue of its higher lipophilicity, independently of the megalin-DBP pathway. Human parathyroid chief cells constitutively express CYP27A1 alongside CYP27B1, providing enzymatic capacity for sequential intracellular 25-hydroxylation and 1α-hydroxylation of cholecalciferol to calcitriol within the parathyroid cell itself (60, 61). If operative, this direct intracellular pathway would represent a route for parathyroid intracrine vitamin D activation that bypasses both impaired hepatic conversion and impaired megalin-dependent uptake. The quantitative contribution of this pathway *in vivo*, however, remains to be established in prospective studies.

The mechanistic basis of the ≥50 ng/ml 25(OH)D threshold requires critical scrutiny. Delivery rate rather than absolute concentration appears to be a primary determinant of PTH suppression: bolus intravenous calcifediol produced abrupt increases in serum 25(OH)D and calcitriol with 40-fold induction of renal CYP24A1 and 13-fold induction of parathyroid CYP24A1, yet achieved only minimal PTH reduction in CKD patients, whereas modified-release oral calcifediol raised 25(OH)D gradually without appreciable CYP24A1 induction and produced meaningful sustained PTH suppression at tenfold lower cumulative exposure in the rat model (62). The mechanistic interpretation is that rapid substrate delivery triggers a surge in parathyroid CYP27B1-derived calcitriol, which strongly induces parathyroid CYP24A1 and catabolizes locally generated calcitriol before it can fully activate VDR. Whether the ≥50 ng/ml threshold reflects a genuine biological requirement for parathyroid VDR activation or the concentration at which gradual substrate provision exceeds this self-limiting catabolic capacity in a VDR-deficient gland has not been resolved. The primary evidence supporting this target derives almost exclusively from investigators affiliated with OPKO Health, the manufacturer of extended-release calcifediol (Rayaldee), and has not been independently replicated.

### 3.3 System-level effects beyond parathyroid regulation

Beyond its direct effects on PTH, vitamin D status is linked to multiple components of the CKD-MBD network, including calcium handling and endocrine regulation. VDR signaling interacts with pathways related to inflammation, oxidative stress, and fibrosis, suggesting broader systemic effects (35). In CKD, deficiency of 25(OH)D is common and has been associated with abnormalities in calcium balance and dysregulation of the FGF-23–Klotho axis (63).

Repletion of 25(OH)D through NVD may help stabilize aspects of this network. Clinical and meta-analytic data suggest that supplementation modestly lowers PTH and may improve surrogate markers of mineral metabolism, generally without frequent hypercalcemia or hyperphosphatemia at standard doses (52). Compared with active vitamin D analogs, NVD alone appears to have largely neutral effects on FGF-23 and mineral load, although results are not entirely consistent (64). When combined with calcimimetic therapy, however, this profile shifts toward reduction in FGF-23, reflecting integrated regulation across complementary pathways.

These findings suggest that NVD functions as a modulator of the CKD-MBD milieu rather than a direct driver of mineral perturbation, although its impact on long-term clinical outcomes remains uncertain.

## 4 Mechanistic integration of PTH control

The therapeutic challenge in SHPT lies not only in reducing PTH levels but in stabilizing coordinated PTH regulation within a physiologically appropriate range, recognizing that the hysteretic nature of SHPT and the irreversible loss of renal mass preclude full restoration of normal physiological regulatory control through biochemical suppression alone. Combination therapy with calcimimetics and NVD provides a framework for integrating secretory and transcriptional control, shifting treatment from isolated pathway suppression toward coordinated regulation. This model emphasizes interaction between regulatory pathways rather than independent axis targeting, aligning therapeutic intervention with the underlying biology of SHPT.

### 4.1 Stabilization of PTH secretion via CaSR activation

Calcimimetics enhance CaSR sensitivity to extracellular calcium, lowering the calcium set-point for PTH secretion and producing rapid reductions in circulating PTH levels (65). In dialysis populations, PTH levels may decrease within 1–2 h after administration, reflecting the immediate effect of CaSR activation on secretory control (66). Clinical studies and meta-analyses demonstrate consistent reductions in both PTH and serum calcium, indicating that calcimimetics drive PTH reduction through stabilization of the secretory component of PTH regulation (39, 40).

This mechanism enables rapid and sustained lowering of PTH while stabilizing short-term fluctuations via calcium-dependent feedback. However, it does not directly address transcriptional regulation of PTH. CaSR activation is also associated with reductions in serum calcium and may induce hypocalcemia, which can trigger counter-regulatory PTH stimulation (42). In addition, calcimimetic therapy has been associated with reduced circulating calcitriol and does not correct the underlying deficiency of 25(OH)D in CKD (42). Gastrointestinal adverse effects, including nausea and vomiting, are also significantly more frequent with calcimimetics than with active vitamin D analogs (48, 49). These observations indicate that while CaSR activation provides effective and immediate control of PTH secretion, it remains insufficient to restore coordinated regulation when used in isolation.

### 4.2 Restoration of PTH transcription via VDR signaling

VDR-mediated signaling provides a transcriptional mechanism for suppressing PTH synthesis. Both active vitamin D compounds and vitamin D prohormones, including 25(OH)D_3_ and 1α-hydroxylated prodrugs such as doxercalciferol, reduce PTH transcription in a VDR-dependent manner, consistent with local intracrine conversion to calcitriol within parathyroid chief cells prior to VDR activation, rather than through direct VDR binding by the prohormone itself (51). Experimental studies further confirm that 25(OH)D suppresses PTH gene expression in parathyroid explants through this substrate-dependent intracrine mechanism in a strictly VDR-dependent manner (61).

The efficacy of this pathway is constrained by the degree of parathyroid VDR expression, which declines progressively with advancing hyperplasia. LIP-mediated suppression of C/EBPβ-dependent induction of the VDR gene promoter results in up to 80% reduction of VDR mRNA in nodular hyperplasia, limiting the transcriptional response to calcitriol regardless of substrate availability (36). Substrate repletion through NVD alone is therefore insufficient to restore VDR-dependent PTH suppression in advanced nodular disease. Nevertheless, experimental evidence from rat models of SHPT demonstrates that correction of vitamin D deficiency through 25(OH)D availability synergizes with calcitriol—through intracrine CYP27B1-mediated conversion and/or direct VDR-AP occupancy—to induce parathyroid C/EBPβ expression, which counteracts LIP-driven VDR silencing and restores parathyroid responsiveness to low-dose active vitamin D, reducing PTH by 50% and suppressing parathyroid gland enlargement with a potency comparable to phosphate restriction (38). The synergy operates through an enhanced C/EBPβ/LIP ratio rather than through substrate provision alone.

NVD therefore complements calcimimetic therapy not merely by restoring substrate availability, but through a mechanistically distinct contribution to parathyroid C/EBPβ-mediated induction of VDR gene expression that may partially counteract LIP-driven VDR suppression. The extent to which calcimimetics additionally contribute to VDR recovery remains under investigation; available evidence from a single uremic rat study suggests partial restoration, though the causal relationship between VDR recovery and PTH suppression in that model is not fully established (67).

A further constraint on intracrine VDR activation is imposed by CYP24A1, which is constitutively present in parathyroid chief cells and strongly induced by intracellularly generated calcitriol, creating a self-limiting catabolic loop that degrades locally produced calcitriol before it can fully activate VDR. This mechanism is particularly relevant in advanced SHPT, where residual VDR signaling capacity must compete with CYP24A1-mediated calcitriol catabolism within a parathyroid cell where VDR expression is already substantially reduced by the LIP/C/EBPβ pathway. Whether the serum 25(OH)D ≥50 ng/ml target reflects a genuine biological requirement for parathyroid VDR activation or the concentration at which gradual substrate provision finally exceeds this self-limiting catabolic capacity in a VDR-deficient gland has not been resolved, and the primary evidence supporting this target derives almost exclusively from investigators affiliated with OPKO Health, the manufacturer of extended-release calcifediol (Rayaldee), without independent replication (68).

An additional mechanistic layer relevant to the synergy between NVD and calcitriol-based strategies concerns the structural organization of the VDR ligand binding domain. The VDR contains two overlapping ligand binding pockets: the canonical genomic pocket (VDR-GP), occupied by calcitriol and mediating classical gene transcription, and an alternative pocket (VDR-AP), to which 25(OH)D_3_ binds preferentially (69, 70). At higher concentrations, 25(OH)D_3_ can also enter the genomic pocket with lower affinity than calcitriol, which may account for the concentration-dependent CYP24A1 induction observed at supraphysiologic 25(OH)D levels. Within this two-pocket framework, simultaneous occupancy of the VDR-AP by 25(OH)D and the VDR-GP by locally generated calcitriol may produce a VDR conformational state that amplifies transactivation synergistically, a mechanism more complex than simple substrate provision alone. This model may provide an additional structural rationale for why restoration of 25(OH)D to adequate but not supraphysiologic levels may complement rather than compete with intracrine calcitriol generation, and may help explain why delivery rate rather than absolute circulating concentration appears to influence PTH suppression efficiency in the context of the ≥50 ng/ml threshold discussion above. Although this model remains hypothetical and has not been directly validated in parathyroid tissue, it provides a potential framework for future investigation.

**Figure 1:**
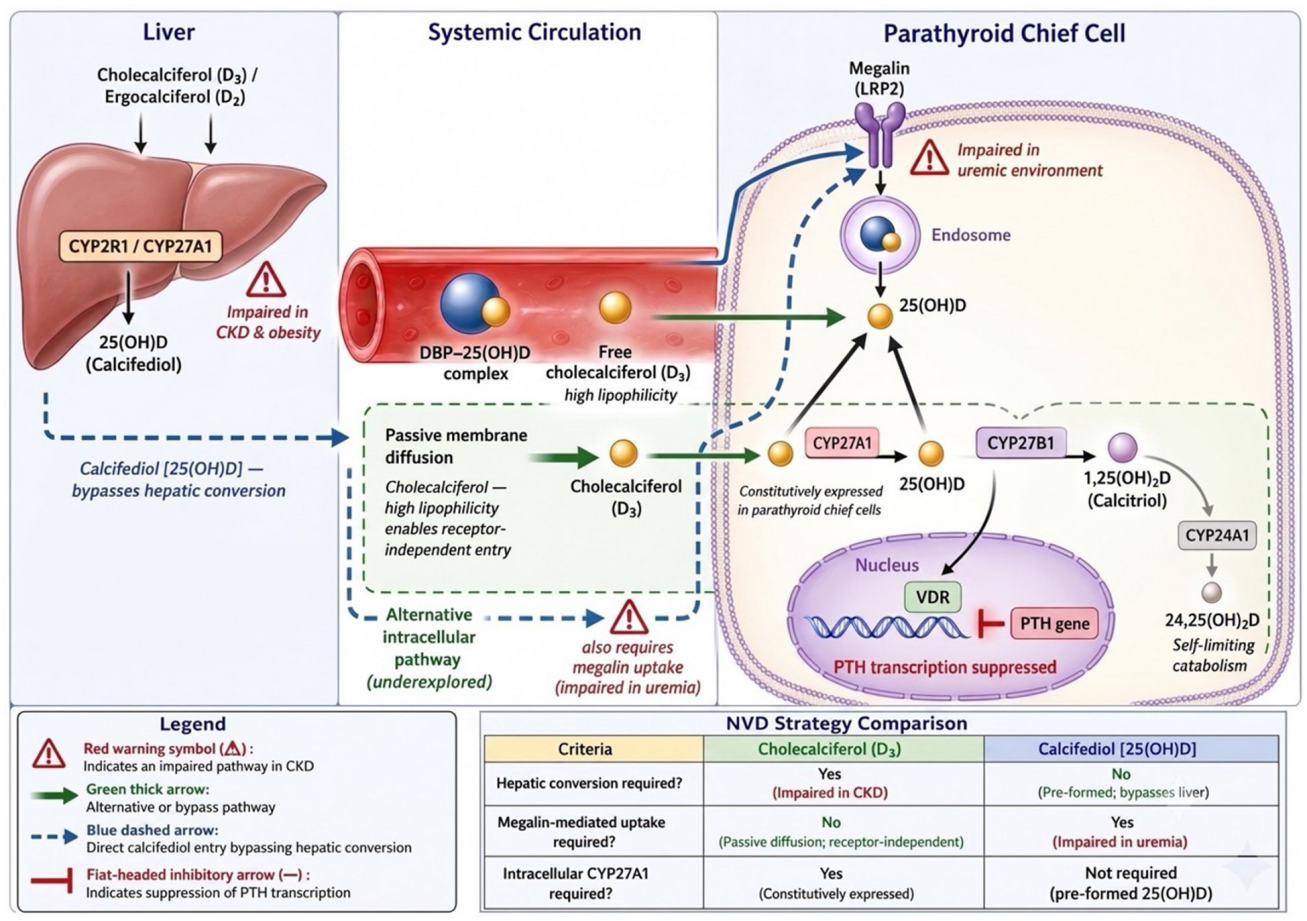
NVD activation pathways, uremic barriers, and the cholecalciferol intracellular bypass route in the parathyroid chief cell. Nutritional vitamin D (cholecalciferol or ergocalciferol) undergoes hepatic 25-hydroxylation via CYP2R1 and CYP27A1 to generate circulating 25(OH)D. This hepatic conversion step is impaired in CKD and obesity. Calcifediol, as a pre-formed 25(OH)D compound, bypasses this barrier and enters the systemic circulation directly, but remains dependent on megalin (LRP2)-mediated endocytosis for parathyroid cellular entry—a route that is likewise hypothesized to be impaired in the uremic environment, by analogy with the well-documented reduction of megalin function in proximal tubular cells. Cholecalciferol, by contrast, may enter parathyroid chief cells directly via passive membrane diffusion, independently of the megalin–DBP pathway, by virtue of its higher lipophilicity. Human parathyroid chief cells constitutively express CYP27A1 alongside CYP27B1, enabling sequential intracellular 25-hydroxylation and 1α-hydroxylation of cholecalciferol to calcitriol within the cell itself—an alternative intracellular activation route that bypasses both uremic barriers. Intracellularly generated calcitriol binds VDR and suppresses PTH gene transcription. CYP24A1-mediated conversion to 24,25(OH)_2_D represents a self-limiting catabolic loop. This proposed intracellular bypass pathway remains hypothetical and has not been directly demonstrated in human parathyroid tissue. Represents an extrapolation from proximal tubular cell data; direct evidence in parathyroid chief cells is currently lacking. CKD, chronic kidney disease; DBP, vitamin D-binding protein; LRP2, low-density lipoprotein receptor-related protein 2; 25(OH)D, 25-hydroxyvitamin D; 1,25(OH)_2_D, 1,25-dihydroxyvitamin D (calcitriol); CYP2R1, vitamin D 25-hydroxylase; CYP27A1, sterol 27-hydroxylase; CYP27B1, 1α-hydroxylase; CYP24A1, 24-hydroxylase; VDR, vitamin D receptor; PTH, parathyroid hormone; NVD, nutritional vitamin D.

The choice of NVD formulation may further modulate the efficiency of this intracrine pathway. Cholecalciferol's higher lipophilicity may support receptor-independent parathyroid cell entry via passive membrane diffusion, bypassing the megalin-dependent uptake mechanism that is hypothesized to be impaired in the uremic environment, by analogy with the well-documented reduction of megalin function in proximal tubular cells; direct evidence in parathyroid chief cells is currently lacking. Calcifediol, although bypassing the hepatic conversion step, remains dependent on megalin-mediated endocytosis for cellular entry, a route that is likewise hypothesized to be impaired in the uremic environment on the same basis (Figure 1).

### 4.3 Reversal of parathyroid resistance

Both CaSR and VDR are downregulated in advanced SHPT, contributing to reduced responsiveness of the parathyroid gland to calcium and vitamin D signaling (71). Experimental studies indicate that these pathways are functionally interconnected: VDR activation can increase CaSR expression, while CaSR signaling may influence VDR expression and activity, suggesting bidirectional crosstalk (72, 73).

Calcimimetic therapy has been shown to increase both CaSR and VDR expression in hyperplastic parathyroid tissue in experimental models, potentially enhancing sensitivity to calcium and vitamin D signaling (67, 73). In a uremic rat study, both cinacalcet and evocalcet produced marked upregulation of parathyroid VDR relative to vehicle-treated animals. In this study, evocalcet achieved greater PTH suppression than cinacalcet (PTH 28.7 ± 24.1 vs. 497.4 ± 655.6 pg/ml; *p* < 0.05) and was also associated with numerically higher VDR expression (1.99-fold vs. 1.33-fold of normal controls), while the vehicle group showed markedly reduced VDR expression (0.32-fold of normal). However, this co-occurrence does not establish a causal relationship between VDR recovery and PTH suppression, as the authors themselves acknowledged that the precise mechanism by which calcimimetics upregulate VDR remains unclear (67), and both outcomes may instead reflect differences in the intrinsic potency of CaSR activation between the two agents rather than a VDR-mediated transcriptional effect. CaSR activation raises intracellular calcium, which may suppress parathyroid TGF-α expression through a cell-autonomous mechanism, attenuating LIP synthesis and partially relieving LIP-mediated suppression of C/EBPβ-dependent VDR gene induction. Under this interpretation, VDR recovery is more accurately characterized as a downstream consequence of reduced parathyroid proliferative drive rather than a direct receptor-upregulating action of calcimimetics, and the precise mechanism remains unclear (67).

The available experimental evidence carries an important limitation. The model employed 5/6 nephrectomized rats with diffuse parathyroid hyperplasia, which does not exhibit the nodular component present in more advanced human SHPT (67). The partial VDR recovery demonstrated in this diffuse hyperplasia model has not been shown to be quantitatively sufficient to restore meaningful VDR-dependent PTH suppression in human nodular hyperplasia, where LIP-driven VDR silencing produces up to 80% reduction in VDR mRNA and where persistent nodular hyperplasia with continued receptor downregulation has been documented histologically even after long-term cinacalcet therapy. In summary, available evidence supports the conclusion that calcimimetic therapy is associated with partial VDR upregulation in experimental diffuse hyperplasia models, but does not establish that this effect translates into clinically meaningful VDR-dependent PTH suppression in human SHPT, particularly in advanced nodular disease.

**Figure 2:**
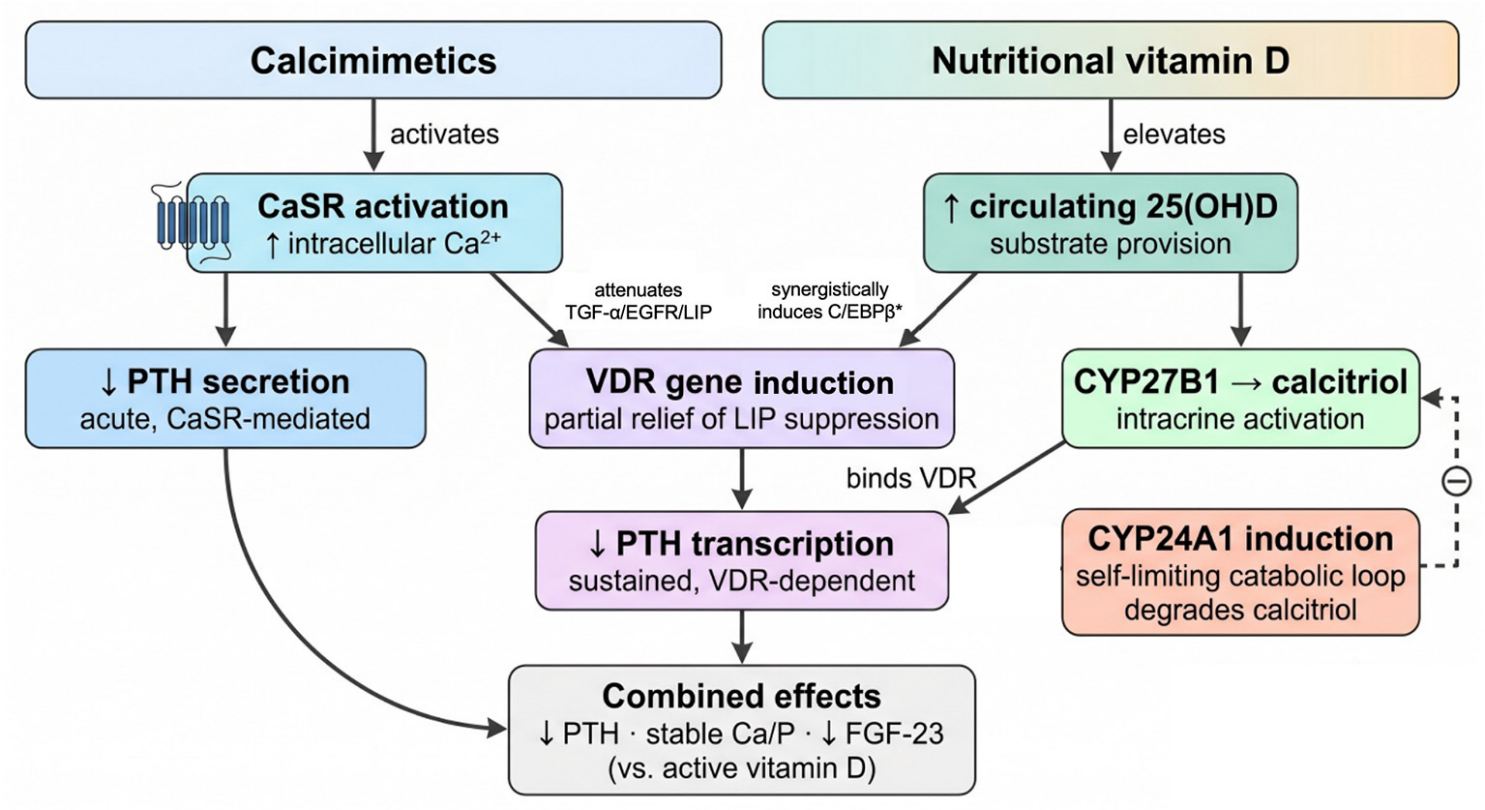
Complementary cellular mechanisms of calcimimetics and nutritional vitamin D in the regulation of PTH in SHPT. Calcimimetics allosterically activate CaSR, lowering the calcium set-point and acutely suppressing PTH secretion. CaSR-mediated elevation of intracellular calcium attenuates parathyroid TGF-α/EGFR signaling, reducing LIP synthesis and partially relieving LIP-mediated suppression of C/EBPβ-dependent VDR gene induction. Nutritional vitamin D increases circulating 25(OH)D, providing substrate for parathyroid CYP27B1-mediated intracrine calcitriol generation; the resulting calcitriol binds VDR and suppresses PTH transcription in a sustained, VDR-dependent manner. In addition, 25(OH)D availability may synergize with calcitriol—through intracrine CYP27B1-mediated conversion and/or direct VDR-AP occupancy—to induce parathyroid C/EBPβ expression, counteracting LIP-driven suppression of C/EBPβ-dependent VDR gene induction through a mechanism complementary to substrate provision. Intracellularly generated calcitriol simultaneously induces CYP24A1, creating a self-limiting catabolic loop that degrades locally produced calcitriol and constrains the efficiency of intracrine VDR activation. Within the combination framework, calcimimetic-mediated attenuation of TGF-α/EGFR/LIP signaling and NVD-mediated C/EBPβ induction converge to support a more favorable C/EBPβ/LIP ratio, complementing both the secretory and transcriptional axes of PTH regulation. *The synergistic induction of parathyroid C/EBPβ by 25(OH)D availability may operate through intracrine conversion to calcitriol via parathyroid CYP27B1, direct occupancy of the alternative VDR binding pocket (VDR-AP) by 25(OH)D itself, or both; the *in vivo* model from which this observation derives (38) cannot dissociate these contributions. SHPT, secondary hyperparathyroidism; PTH, parathyroid hormone; CaSR, calcium-sensing receptor; VDR, vitamin D receptor; NVD, nutritional vitamin D; 25(OH)D, 25-hydroxyvitamin D; TGF-α, transforming growth factor-α; EGFR, epidermal growth factor receptor; LIP, liver-enriched inhibitory protein; C/EBPβ, CCAAT/enhancer-binding protein-β; CYP27B1, 1α-hydroxylase; CYP24A1, 24-hydroxylase.

When combined with restoration of vitamin D substrate availability through NVD, the partial attenuation of LIP-driven VDR suppression by calcimimetics may produce complementary effects at the receptor level, supporting a more favorable C/EBPβ/LIP ratio within the parathyroid chief cell. However, the extent to which this interaction translates into clinically meaningful reversal of functional resistance in human SHPT remains uncertain, and available evidence suggests that such effects are partial rather than complete. These complementary pathways and their convergence within the parathyroid gland are summarized in Figure 2.

## 5 Translating mechanism into clinical phenotypes

The mechanistic framework provides a basis for understanding how therapeutic interventions translate into clinically observable effects. Rather than focusing solely on absolute PTH reduction, clinical responses should be interpreted in terms of biochemical stability and coordinated regulation within the CKD-MBD network. The comparative biochemical profiles and clinical positioning of the principal pharmacologic regimens for SHPT are summarized in Table 1.

**Table 1:** Comparative biochemical and clinical profiles of pharmacologic strategies for SHPT in CKD.

Regimen	PTH	Serum Ca	Serum P	FGF-23	Hypocalcemia risk	Best-suited patient profile
Active vitamin D analog (calcitriol, paricalcitol)	↓↓	↑	↑	↑↑	Low	Mild–moderate SHPT, low calcium/phosphate burden, normal vascular status
NVD alone (cholecalciferol/ergocalciferol)^†^	↓ (modest)	↔/mild ↑	↔/mild ↑	↔	Very low	Early CKD with 25(OH)D deficiency and mild SHPT
Calcimimetic alone (cinacalcet/etelcalcetide)	↓↓	↓↓	↓	↓↓	Moderate–high	Dialysis SHPT with hypercalcemia or vascular calcification concern
Calcimimetic with active vitamin D^‡^	↓*↓↓*	↓	↔/mild ↓	↓/↓↓	Moderate	Severe SHPT requiring maximal PTH suppression; tolerated mineral profile
Calcimimetic with NVD	↓↓ to ↓*↓↓*	mild ↓/↔	↓/↔	↓↓	Low–moderate	SHPT with concurrent 25(OH)D deficiency, vascular calcification, or intolerance to active vitamin D
Triple therapy (calcimimetic with active and NVD)	↓*↓↓*	↔	↔/mild ↑	↑	Moderate	Refractory SHPT prior to consideration of parathyroidectomy

SHPT, secondary hyperparathyroidism; CKD, chronic kidney disease; PTH, parathyroid hormone; Ca, calcium; P, phosphate; FGF-23, fibroblast growth factor 23; NVD, nutritional vitamin D.

Symbols: ↑, increase; ↓, decrease; ↔, no consistent change or stabilization.

^†^The serum 25(OH)D target of ≥50 ng/ml cited in the context of NVD-based PTH suppression derives primarily from studies conducted by OPKO Health-affiliated investigators and has not been independently validated. The mechanistic basis of this threshold remains uncertain.

^‡^Network meta-analytic evidence indicates that calcimimetic-induced reductions in serum calcium are not fully corrected by concurrent active vitamin D analog use. Biochemical improvements with calcimimetic-based regimens have not demonstrated significant benefits on mortality, cardiovascular events, or fractures in randomized controlled trials.

### 5.1 Biochemical stability beyond absolute PTH reduction

Physiological regulation of PTH involves both rapid changes in secretion and slower adjustments in transcription and hormone synthesis. Secretory responses mediated through CaSR can occur within minutes and are often pulsatile, whereas transcriptional regulation through vitamin D–dependent pathways leads to more sustained changes in PTH production (74). These temporal differences indicate that regulation of PTH depends not only on its absolute level but also on stability over time.

Therapeutic strategies that combine rapid suppression of PTH secretion with sustained transcriptional regulation may therefore achieve more stable biochemical control. Evidence from endocrine and modeling studies suggests that reducing short-term fluctuations in PTH, rather than pursuing maximal suppression, is associated with more consistent effects on calcium balance and bone turnover (75, 76). In CKD-related SHPT, this aligns with combination approaches that reduce variability and rebound phenomena observed with single-pathway interventions.

Both excessively high and overly suppressed PTH levels have been associated with adverse skeletal and metabolic consequences. PTH retains important compensatory actions in the CKD setting, including modulation of calcium reabsorption, stimulation of bone turnover, and indirect support of renal 1α-hydroxylase activity, that cannot be fully substituted once renal mass is lost. Observational data across CKD stages demonstrate a U-shaped relationship between PTH levels and adverse skeletal outcomes, with fracture risk, vascular events, and mortality minimized at intermediate PTH levels and rising at both extremes (77). Adynamic bone disease, characterized by markedly reduced bone turnover and associated with fragility and vascular calcification, is strongly linked to PTH oversuppression and represents a recognized complication of aggressive SHPT therapy. The therapeutic goal of combination calcimimetic and NVD therapy should therefore be interpreted as achieving stable PTH regulation within a physiologically appropriate range rather than pursuing maximal PTH suppression as an end in itself.

### 5.2 Mineral balance: interactions between therapeutic effects

Calcimimetics and NVD exert distinct and partially opposing effects on calcium homeostasis. Calcimimetics lower PTH and serum calcium by enhancing CaSR sensitivity and are consistently associated with reductions in serum calcium and calcium–phosphate product in dialysis populations (42, 65). In non-dialysis CKD, these effects may be accompanied by hypocalcemia and less consistent or attenuated reductions in serum phosphate in certain clinical contexts, particularly when used without concurrent supplementation (44).

In contrast, NVD primarily increases circulating 25(OH)D and has relatively modest effects on serum calcium. Meta-analytic data suggest small increases in serum calcium (approximately 0.23 mg/dl) with low rates of hypercalcemia in non-dialysis CKD populations (52, 78). Compared with active vitamin D analogs, NVD alone appears to have largely neutral effects on phosphate and FGF-23, whereas in combination with calcimimetic therapy, this profile shifts toward reduction in FGF-23 (35).

These complementary effects suggest that combination therapy may partially stabilize mineral balance compared with active vitamin D–based strategies. Restoration of vitamin D status through NVD may attenuate the hypocalcemia risk associated with calcimimetics to a modest degree; however, network meta-analytic evidence from 21 RCTs indicates that combining calcimimetics with active vitamin D analogs still produces a net reduction in serum calcium relative to placebo (49), demonstrating that calcium-lowering effects are not fully corrected even with concurrent vitamin D use. Whether NVD exerts a comparably smaller offsetting effect has not been directly established. Direct comparative evidence remains limited, and the assumption that combination calcimimetic and NVD therapy achieves a neutralized calcium profile should not be made without individualized monitoring.

### 5.3 Endocrine network effects: FGF-23 and CKD-MBD signaling

FGF-23 is a central component of the CKD-MBD endocrine network, interacting with PTH, phosphate, and vitamin D metabolism to regulate mineral homeostasis. As kidney function declines, FGF-23 levels rise early to maintain phosphate balance but may become markedly elevated in advanced CKD, and higher levels have been associated with cardiovascular complications and mortality in observational studies (25, 26).

Therapies for SHPT exert differential effects on FGF-23. Active vitamin D analogs consistently increase FGF-23 levels, whereas NVD generally has neutral or smaller effects while still lowering PTH in many cases (79). Calcimimetics are generally associated with reductions in PTH and serum calcium, and are also associated with reductions in FGF-23, although the magnitude may vary across clinical contexts (26).

These differences suggest that combination approaches may allow effective PTH control with concurrent reduction in FGF-23, rather than the increases observed with active vitamin D–based strategies. However, whether modulation of FGF-23 translates into improved clinical outcomes remains uncertain.

A clinically important and counterintuitive aspect of FGF23 biology in CKD concerns its relationship with vitamin D catabolism. Although FGF23 potently induces renal CYP24A1 in physiological conditions, thereby promoting degradation of both 25(OH)D and 1,25(OH)_2_D, clinical data reveal the opposite systemic outcome in CKD. In the Seattle Kidney Study, serum 24,25(OH)_2_D, the primary product of CYP24A1-mediated 25(OH)D catabolism and a validated biomarker of overall vitamin D catabolic activity, declined progressively with decreasing estimated glomerular filtration rate (eGFR), from 3.6 ng/ml at eGFR ≥60 to 1.7 ng/ml at eGFR < 15 ml/min/1.73m^2^, despite concurrent dramatic elevation of FGF23 (80). FGF23 was not independently correlated with 24,25(OH)_2_D in unadjusted analyses, and lower eGFR remained the dominant predictor of reduced 24,25(OH)_2_D after full adjustment. These findings indicate that the progressive reduction in functional renal mass and proximal tubular metabolic capacity, rather than FGF23-mediated CYP24A1 induction, is the primary determinant of the CKD vitamin D catabolic phenotype, a conclusion corroborated by parallel observations in human and mouse CKD models (81). Clinically, this means that CKD represents a state of stagnant rather than accelerated vitamin D catabolism: both 1,25(OH)_2_D production and 25(OH)D catabolism are simultaneously reduced. This observation has direct therapeutic relevance for NVD supplementation: the impaired catabolism implies that supplemented 25(OH)D is not rapidly degraded by FGF23-driven CYP24A1 activity, and substrate availability remains the operative rate-limiting factor for both endocrine and intracrine vitamin D activation across CKD stages.

### 5.4 Clinical implications: toward physiological alignment

The downstream effects of PTH dysregulation extend beyond the parathyroid gland to skeletal and cardiovascular systems, reflecting the integrated nature of CKD-MBD. Experimental studies show that elevated PTH and phosphate contribute to bone loss and vascular calcification, and that normalization of PTH alone does not fully prevent vascular injury (28). The coexistence of bone demineralization and vascular calcification further highlights the systemic impact of mineral dysregulation (82, 83).

However, the relationship between PTH suppression and skeletal outcomes in CKD is complex and non-linear, and reduction of circulating PTH should not be implicitly equated with improved bone health. Observational data demonstrate a U-shaped association between PTH levels and adverse skeletal outcomes across CKD stages, with fracture risk, vascular events, and mortality minimized at intermediate PTH levels and rising at both extremes (77, 84). Markedly elevated PTH drives high-turnover cortical bone loss and osteodystrophy, whereas excessively suppressed PTH promotes adynamic bone disease (ABD), characterized by severely reduced osteoblast and osteoclast activity, impaired bone repair capacity, increased fracture risk, and promotion of vascular calcification, a recognized complication of aggressive SHPT therapy (85, 86).

Furthermore, biochemical control of PTH does not necessarily equate to restoration of normal bone turnover. CKD introduces skeletal resistance to PTH through multiple mechanisms, including downregulation of parathyroid hormone type 1 receptor (PTH1R) expression, suppression of Wnt/β-catenin signaling, elevated sclerostin, and uremic toxin-mediated impairment of osteoblast function, that persist independently of circulating PTH levels (87, 88). Experimental evidence further illustrates this dissociation: calcitriol-mediated PTH suppression of approximately 60% in a CKD animal model did not improve bone structure or mechanical properties, raising doubts about the skeletal benefit of PTH suppression alone (89).

Within this context, therapeutic strategies that stabilize PTH while avoiding excessive calcium and phosphate loading may better align with physiological regulation. Calcimimetics lower PTH without increasing calcium–phosphate burden and may improve markers of bone turnover and calcification-related surrogates such as calciprotein particles (90). However, most evidence is derived from experimental models or surrogate endpoints rather than definitive clinical outcomes (91).

These findings suggest that the clinical value of combination therapy lies in maintaining PTH within a physiologically appropriate range, avoiding both the skeletal consequences of sustained PTH excess and the ABD risk of excessive suppression, rather than achieving maximal PTH reduction as a therapeutic endpoint in itself.

## 6 Mechanism-informed therapeutic positioning

Therapeutic strategies in SHPT can be more effectively interpreted when aligned with the underlying regulatory defects of PTH. Rather than viewing available treatments as interchangeable tools for lowering PTH, a mechanism-informed approach emphasizes matching therapeutic actions to specific disturbances in mineral and endocrine regulation. Within this framework, NVD can be positioned as a substrate-level intervention that complements calcimimetic therapy. By restoring 25(OH)D availability, NVD supports vitamin D–dependent transcriptional control and may help address limitations of secretion-focused therapy.

### 6.1 Reframing treatment strategies through a mechanism-based lens

Current treatment strategies for SHPT can be better understood when mapped to their primary mechanisms of action. Calcimimetic monotherapy targets PTH secretion by enhancing CaSR sensitivity, producing rapid reductions in circulating PTH and often decreasing serum calcium (65, 92). However, this approach does not restore transcriptional regulation and may be associated with hypocalcemia and reduced calcitriol levels, reflecting incomplete regulatory control. Combination therapy with calcimimetics and active vitamin D analogs engages both secretion and synthesis pathways and generally achieves greater PTH reduction than monotherapy. However, this approach is frequently associated with increases in serum calcium, phosphate, and FGF-23 due to systemic vitamin D effects (93). In contrast, combining calcimimetics with NVD represents a distinct strategy that complements secretory control with substrate-dependent support of transcriptional regulation. NVD has modest and generally neutral effects on calcium, phosphate, and FGF-23, and is associated with a reduction in FGF-23 when combined with calcimimetic therapy, although the magnitude may vary across clinical settings (79). Although direct evidence remains limited, this approach may support PTH control while maintaining a more stable mineral profile.

Translated into a temporal therapeutic framework, the dual-axis model supports stage-specific intervention targets that may meaningfully alter disease trajectory when applied at the appropriate disease phase. In the pre-hysteretic phase of early CKD, both regulatory axes retain functional integrity: CaSR responsiveness to extracellular calcium is preserved, and VDR-dependent transcriptional suppression of PTH remains operative. During this window, NVD supplementation addresses the substrate-level limitation of the VDR axis while simultaneously supporting CaSR gene expression through vitamin D response elements, potentially delaying the progressive receptor downregulation that drives treatment resistance in advanced disease. Phosphate control during this phase targets the upstream driver of parathyroid TGF-α induction and LIP-mediated VDR silencing, providing a mechanistic rationale for early dietary and pharmacological phosphate management as a disease-modifying strategy rather than merely a biochemical target.

As SHPT advances into the post-hysteretic phase, progressive nodular hyperplasia and receptor downregulation shift the therapeutic emphasis from preservation of regulatory axis integrity toward stabilization of a dysregulated steady state. Calcimimetics address the CaSR axis directly, while NVD supports residual VDR function through C/EBPβ-mediated attenuation of LIP-driven promoter suppression. The combination therefore engages both axes in a manner that is mechanistically adapted to the disease stage, representing stage-appropriate dual-axis engagement within the constraints imposed by established parathyroid remodeling.

### 6.2 Phenotype-guided therapeutic alignment

An important implication of this framework is that SHPT management should be guided by patient-specific phenotypes rather than uniform biochemical targets. CKD-MBD is heterogeneous, with variation in calcium balance, phosphate levels, vitamin D status, and bone turnover, and current guidelines emphasize individualized management, with PTH targets representing pragmatic clinical benchmarks rather than physiologically validated endpoints (94).

In patients with hypercalcemia or elevated calcium–phosphate burden, therapies that avoid additional mineral loading are preferred. Active vitamin D analogs can increase calcium and phosphate, whereas calcimimetics reduce both PTH and serum calcium and have been shown to lower calcium–phosphate product in meta-analyses (42, 92). In this setting, combining calcimimetics with NVD may provide PTH control while maintaining relatively stable mineral balance, as NVD has more modest effects on calcium, phosphate, and FGF-23 (79).

Conversely, patients with hypocalcemia or low bone turnover require caution, as excessive suppression of PTH may be harmful. Low-turnover states, including ABD, have been associated with aggressive suppression of PTH and may impair bone remodeling and promote extra-skeletal calcification (86). Careful titration and avoidance of overt suppression are therefore recommended.

Patients with significant 25(OH)D deficiency represent a distinct subgroup in whom NVD plays a central role. Vitamin D deficiency is common in CKD and contributes to SHPT; supplementation reliably increases 25(OH)D levels with modest effects on PTH and minimal effects on phosphate (52, 79).

### 6.3 Stage-specific therapeutic considerations across CKD

The therapeutic positioning of calcimimetic-based combination therapy reflects the stage-dependent nature of CKD-MBD progression. CKD-MBD progresses through two functionally distinct phases that require different therapeutic emphases. In the pre-hysteretic phase, corresponding broadly to CKD stages 1–3 and early stage 4 before nodular parathyroid hyperplasia and progressive CaSR and VDR downregulation are established, the regulatory system retains sufficient plasticity that timely intervention targeting the underlying drivers of parathyroid stimulation may stabilize or attenuate SHPT progression. During this window, correction of 25(OH)D deficiency through NVD supplementation, phosphate control, and avoidance of persistent hypocalcemia represent the primary therapeutic tools. Notably, FGF-23 elevation precedes detectable PTH rise in early CKD (95, 96), and early phosphate control has been shown to attenuate both PTH and FGF-23 elevation even in normophosphatemic CKD patients (97), supporting the concept that upstream intervention in the pre-hysteretic phase may modify disease trajectory.

In contrast, calcimimetic-based strategies primarily address the post-hysteretic phase of established SHPT, corresponding broadly to late stage 4 and stage 5 CKD including dialysis-dependent patients, characterized by parathyroid hyperplasia, progressive receptor downregulation, and a dysregulated steady state that is refractory to correction through substrate provision or calcium signaling alone. Within this framework, NVD in the combination regimen serves a complementary mechanistic role: it supports residual intracrine vitamin D signaling, contributes to C/EBPβ-mediated attenuation of LIP-driven VDR suppression, and maintains substrate availability for parathyroid chief cells operating under conditions of impaired megalin-dependent uptake.

These stage-dependent distinctions are reflected in current clinical practice across the CKD continuum. In non-dialysis CKD, correction of 25(OH)D deficiency is recommended as an early strategy, while active vitamin D analogs are typically reserved for more progressive SHPT (45, 98). NVD reliably increases 25(OH)D levels, but its effect on PTH reduction is modest and variable (79).

In dialysis populations, loss of renal 1α-hydroxylase shifts PTH control toward calcimimetics and active vitamin D analogs (34). However, 25(OH)D deficiency remains common, and NVD or calcifediol may still provide substrate for extrarenal vitamin D activation (99). A pilot randomized trial in hemodialysis patients showed that extended-release calcifediol increased 25(OH)D and 1,25(OH)_2_D and stabilized PTH without inducing hypercalcemia or hyperphosphatemia (100).

These observations suggest that NVD functions as a supportive therapy across CKD stages. In early CKD, it may help attenuate SHPT progression, whereas in dialysis populations it may complement other therapies by maintaining substrate availability. Current evidence does not support NVD as a stand-alone treatment in advanced SHPT.

## 7 Clinical application of combination therapy

The practical application of combination therapy requires a flexible and iterative approach that reflects the dynamic nature of SHPT. Rather than adhering to rigid algorithms, treatment should be adjusted according to evolving biochemical parameters and clinical context.

### 7.1 Initiation: identifying incomplete control

Initiation or optimization of NVD should be considered when SHPT remains incompletely controlled in the setting of low 25(OH)D, rather than immediately escalating calcimimetic or active vitamin D therapy. In non-dialysis CKD, monitoring of PTH from stage 3 onward and assessment of modifiable drivers such as vitamin D deficiency, hyperphosphatemia, and hypocalcemia are recommended before intensifying treatment (98). Low 25(OH)D with persistently elevated or rising PTH may indicate inadequate substrate availability, although NVD alone usually produces modest and variable PTH reduction (99).

The effect of NVD appears greater when deficiency is adequately corrected, although the threshold for meaningful PTH reduction remains uncertain. The ≥50 ng/ml target cited in some calcifediol-based literature has not been independently validated and derives primarily from investigators affiliated with the manufacturer of extended-release calcifediol, as discussed in Sections 3.2 and 4.2; its clinical utility as a treatment target therefore remains unestablished. Regardless of the specific threshold pursued, NVD more reliably raises 25(OH)D than lowers PTH, and its role is best viewed as supportive rather than definitive therapy.

Initiation should be individualized according to CKD stage, baseline calcium and phosphate, current therapy, and prior response. In patients receiving calcimimetics, correction of low 25(OH)D may support vitamin D–dependent signaling, but does not replace established PTH-lowering treatment (45, 99).

### 7.2 Titration: coordinating therapeutic effects

Titration in combination therapy should reflect the complementary effects of calcimimetics and NVD. Calcimimetics act on CaSR to suppress PTH secretion and are typically titrated stepwise (e.g., 30–180 mg/day for cinacalcet) to achieve pragmatically defined PTH target ranges while minimizing fluctuations (101, 102). In parallel, NVD is adjusted to restore and maintain adequate 25(OH)D levels, supporting VDR-mediated regulation and potentially enhancing treatment response (52, 103).

Clinical studies suggest that combination therapy allows lower effective doses of each component. In algorithm-based approaches, calcimimetic initiation is often accompanied by a 20%−50% reduction in active vitamin D dose while maintaining biochemical control (102). Addition of NVD has been associated with further PTH reduction and improved 25(OH)D status without increased risk of hypo- or hypercalcemia (104). However, the study by Zheng et al. (104) included a mandatory 30-day calcitriol washout before enrollment, followed by concurrent initiation of cinacalcet, calcitriol, and cholecalciferol in both treatment groups. The greater reduction in PTH observed in the cholecalciferol group therefore reflects the incremental effect of NVD within a triple-combination regimen comprising cinacalcet, calcitriol, and cholecalciferol. This reference is therefore cited as support for a triple-combination strategy.

Dose adjustment should be guided by longitudinal trends in PTH, calcium, phosphate, and 25(OH)D rather than isolated measurements (34, 105). Vitamin D deficiency during calcimimetic therapy has been associated with worse outcomes despite similar PTH levels, supporting maintenance of adequate 25(OH)D (106).

NVD dose selection should be guided by CKD stage, given the fundamentally different safety profiles across the disease continuum. In CKD stages 3–4, residual renal CYP27B1 activity means that supplemented 25(OH)D can still be converted to calcitriol endogenously; higher NVD doses may increase susceptibility to hypercalcemia and hyperphosphatemia, particularly when combined with calcimimetics whose calcium-lowering effect may mask early biochemical changes. In this setting, doses consistent with current Kidney Disease: Improving Global Outcomes (KDIGO) guidance (800–2,000 IU/day of cholecalciferol or ergocalciferol) represent a reasonable starting point, with upward adjustment guided by serial monitoring of serum calcium, phosphate, and 25(OH)D. In CKD stage 5D, residual renal 1alpha-hydroxylation is negligible, substantially altering the risk profile; higher doses have been used in supporting clinical trials conducted in dialysis patients and were well-tolerated in that context, but should not be extrapolated to earlier CKD stages without appropriate caution (104). Tolerability data from Zheng et al. derive from hemodialysis patients receiving cholecalciferol 5,000 IU/day co-initiated with cinacalcet and calcitriol following a pre-enrollment calcitriol washout, and should be interpreted within that specific clinical context.

When NVD is initiated alongside calcimimetic therapy, serum calcium, phosphate, and 25(OH)D should be monitored regularly during the first 3 months of treatment (e.g., every 4–8 weeks), with less frequent monitoring once biochemical stability has been achieved (63, 98). Maintenance of 25(OH)D concentrations within generally accepted sufficiency ranges may be considered to guide dose adjustment, while acknowledging that the optimal target range for NVD-calcimimetic combination therapy has not been established in prospective clinical trials (52, 63).

### 7.3 Monitoring: evaluating treatment stability

Monitoring should extend beyond PTH to include calcium, phosphate, and 25(OH)D. Stable patients are typically assessed every 3–12 months, with more frequent monitoring during treatment initiation or dose adjustment (98). Maintenance of adequate 25(OH)D is recommended even with active vitamin D therapy (63). Renal function and urinary calcium may also be monitored to detect treatment-related complications (98).

Closer monitoring is required during early treatment phases, when biochemical fluctuations are more common (98). Transient increases in serum calcium may occur following therapy, with peak levels observed several hours after administration in some settings (102). These findings support interpretation based on trends rather than single measurements.

Bone turnover markers such as procollagen type 1 N-terminal propeptide (P1NP) and C-terminal telopeptide of type I collagen (CTX) may provide additional insight into skeletal response, particularly in low-turnover states (107, 108). Although not routinely required, they may be considered for evaluating treatment-related changes in bone metabolism.

### 7.4 Managing imbalance: avoiding overcorrection

Hypocalcemia is the most common adverse effect of calcimimetic therapy, with meta-analyses reporting increased risk and incidence up to ~60% in non-dialysis CKD (109, 110). Most cases are mild and can be managed conservatively (40). Initial management includes dietary calcium adjustment, dialysate modification, or supplementation, while more severe cases may require dose reduction or therapy adjustment (40, 44).

Avoidance of excessive PTH suppression is equally important, as this may lead to low-turnover bone disease (42). Experimental and clinical observations further show that allowing PTH to recover from very low levels, such as through lowering dialysate calcium, can restore bone formation and reverse ABD features (111).

Management should therefore focus on avoiding both hypocalcemia and excessive suppression. Treatment adjustment should be individualized, with monitoring of calcium, phosphate, and PTH trends (112).

## 8 Future directions: from mechanism to clinical outcomes

Despite a coherent mechanistic rationale linking dysregulated PTH control to SHPT pathophysiology, current evidence remains largely centered on biochemical endpoints rather than patient-centered outcomes. Most studies demonstrate improvements in PTH, calcium, and phosphate control; however, the extent to which these changes translate into reductions in fracture risk, cardiovascular events, or mortality remains uncertain (34). This gap highlights the need for future research to move beyond surrogate markers and establish whether restoring coordinated regulation confers meaningful clinical benefit.

An additional unresolved issue is the optimal role and target range of 25(OH)D. While deficiency is associated with worse biochemical control and adverse outcomes in CKD, the threshold required for effective restoration of transcriptional regulation remains unclear and may vary by disease stage (52). In early CKD, higher 25(OH)D levels may support residual calcitriol production, whereas in dialysis populations, potential benefits are more likely mediated through extra-renal or intracrine pathways. Clarifying stage-specific targets is therefore important for optimizing the use of NVD.

A further evidence gap warranting explicit acknowledgment concerns the meta-analytic evidence base for the calcimimetic-plus-NVD combination specifically. The meta-analyses cited in this manuscript predominantly examined calcimimetics combined with active vitamin D analogs—calcitriol, paricalcitol, or other 1α-hydroxylated compounds—rather than with cholecalciferol or ergocalciferol. Dedicated meta-analyses specifically examining the combination of calcimimetics with nutritional vitamin D are largely absent from the published literature. The clinical evidence base for this combination therefore currently rests on a small number of individual trials rather than a body of meta-analytic evidence comparable to that supporting calcimimetics with active vitamin D. Future studies should address this gap directly, ideally through adequately powered randomized controlled trials and subsequent meta-analyses that treat NVD as a distinct intervention category rather than grouping it with active vitamin D analogs.

Future studies should incorporate mechanism-informed designs that align therapeutic interventions with underlying regulatory disturbances. Stratification based on calcium balance, PTH dynamics, and vitamin D status—potentially complemented by biomarkers such as FGF-23 and Klotho—may help identify subgroups most likely to benefit from combination therapy. Emerging approaches, including extended-release vitamin D formulations and next-generation calcimimetics, may offer improved pharmacologic stability and tolerability. Overall, these directions support a shift toward integrated, physiology-informed treatment strategies, although robust outcome-based evidence remains a key unmet need.

## 9 Conclusion

SHPT in CKD is characterized not only by elevated PTH levels but also by loss of coordinated endocrine regulation across interconnected mineral pathways. In this context, combination therapy with calcimimetics and NVD offers a complementary strategy that addresses both rapid hormonal responses and longer-term regulatory processes. Rather than focusing solely on guideline-defined PTH targets, which represent pragmatic clinical thresholds rather than physiologically derived optima, this approach emphasizes stabilization of biochemical dynamics and maintenance of system-level balance within the CKD-MBD network, characterized by substantial reduction in PTH, stabilization or mild reduction of calcium and phosphate, and concurrent reduction in FGF-23. Clinically, this perspective supports a shift from pathway-specific suppression toward integrated management of mineral and endocrine disturbances. However, evidence supporting this strategy remains predominantly biochemical, and key uncertainties persist regarding optimal treatment targets, patient selection, and effects on clinically meaningful outcomes.
